# An observational study to assess changes in social inequality in smoking-attributable upper aero digestive tract cancer mortality among Canadian males between 1986 and 2001

**DOI:** 10.1186/1471-2458-13-328

**Published:** 2013-04-10

**Authors:** Sonica Singhal, Carlos R Quiñonez, Prabhat Jha

**Affiliations:** 1 Community Dental Health Services Research Unit, Discipline of Dental Public Health, Faculty of Dentistry, University of Toronto, Toronto, ON M5G 1G6, Canada; 2 Centre for Global Health Research, Li Ka Shing Knowledge Institute, St. Michael’s Hospital, Toronto, Canada; 3 Dalla Lana School of Public Health, University of Toronto, Toronto, Canada

**Keywords:** Smoking, Upper aero-digestive tract cancer, Socioeconomics, Mortality

## Abstract

**Background:**

Tobacco and low socioeconomic status have been acknowledged as potential risk factors for upper aero-digestive tract (UADT) cancers in North America. In context of reducing adult male smoking prevalence (by over 50%), in the past few decades in Canada, this study tried to document changes in smoking-attributable UADT cancer mortality rates, among Canadian males of different social strata, between 1986 and 2001.

**Methods:**

The contribution of smoking to UADT cancer mortality was estimated indirectly by using lung cancer mortality as an indicator of the accumulated mortality from smoking in a population. This method was applied to UADT cancer death rates of 35–69 year old socially stratified males. Data, stratified by neighborhood income quintile, could be obtained from Statistics Canada, for four census years, 1986, 1991, 1996, and 2001.

**Results:**

A total of 2704 male deaths were analyzed. Between 1986 and 2001, UADT cancer deaths reduced by 30% (32 to 22 per 100,000) but the proportion of these deaths attributable to smoking reduced much more, by 41% (22 to 13 per 100,000). In the span of fifteen years, absolute social inequality (measured by rate difference between the highest and the lowest stratum) in smoking-attributable male UADT cancer mortality in Canada reduced by 47% and relative social inequality (measured by rate ratios) reduced by 9%.

**Conclusion:**

The present analyses reveal that between 1986 and 2001, smoking-attributable UADT cancer mortality rates among adult males (35–69 years) in Canada reduced in all social strata and the social inequalities in these rates have narrowed. Analysis of more current data will be of interest to confirm these trends.

## Background

The sixth most common cancers, to occur worldwide, are the cancers of upper aero-digestive tract (UADT), accounting for approximately five percent of all malignancies [1]. The poor prognosis [2], occurrence of additional cancers of the same or related sites [3], a low five-year survival rate (overall approximately 64% with variation depending on the site) [4], and the economic burden to the health care system and society [5] make such cancers a serious public health concern. The burden of these cancers is more prevalent in males; a study looking at the trends of these cancers in Ontario, Canada, found UADT cancers to be three times more prevalent in males as compared to females and this ratio remained constant from 1984 to 2001 [6]. Tobacco [7-10] and low socioeconomic status [2,11-13] have been acknowledged as risk factors for UADT cancers; approximately, 65% of all UADT cancers are attributable to smoking [14].

The prevalence of smoking in adult males has decreased by over fifty percent in the past few decades in high-income countries, including Canada and the US [15], but most of these countries have observed the greatest declines among higher social strata [16,17]. Concern arises if these declines in smoking prevalence have similar or different effects on smoking-attributable mortality rates for males of different social strata [17]. In the US, death rates due to cancer of the mouth and pharynx fell from 1993 to 2007, but the declines were largely limited to those with higher educational attainment [12]. We know of no similar study in Canada; specifically, there has been no study that quantifies changes in UADT cancer mortality attributable to smoking in different social strata. Thus the aim of this paper is to describe changes in smoking-attributable UADT cancer mortality among Canadian males of different social strata between 1986 and 2001.

## Methods

The approximate contribution of smoking to UADT cancer mortality among Canadian males was estimated indirectly by a method, developed by Peto et al. [18], which has been adapted to analyze social-stratum specific death rates from smoking [19]. This method uses lung cancer mortality as an indicator of the accumulated mortality from smoking in a population [20], and can therefore be used to determine the proportions of smoking-attributable mortalities from other smoking-related diseases, such as UADT cancers [21].

We applied these methods to UADT cancer death rates of 35–69 year old Canadian males of urban Canada (census metropolitan areas, which constitute 60% of male population of Canada), for four census years; 1986, 1991, 1996, and 2001. We obtained 5-year age, sex, and disease-specific mortality data for different social strata, for the four census years, from Statistics Canada. We also obtained population count for the four respective years. These data at Statistics Canada are collected from the Canadian Mortality Data Base and population census, respectively. Data included populations living in institutions as well and were satisfactorily complete. Data obtained were socially stratified by neighborhood income quintile [20], which is determined by the percentage of population in their neighbourhood below the low-income cut-offs [20]. Quintiles of population were ranked from the lowest to the highest as: poorest, poorer, average, richer, and richest, respectively. The use of neighborhood level information was considered reasonable as past studies have argued for the validity of using neighborhood income quintiles as a proxy for individual socioeconomic status [20,22]. Metropolitan areas were used because neighborhoods are more clearly defined and residential segregation by income is more pronounced in big cities than in small towns and rural areas [23].

In the absence of any nationally representative study of smoking and mortality rates, CPS II study (a prospective cohort study of one million Americans, conducted during 1980’s, called the Cancer Prevention Study II) was considered as the reference population, from which lung cancer mortality rates of smokers and never-smokers, and the relative risks for the UADT cancers were considered. For women, as the consequences of smoking epidemic were still to develop in 1980’s [24,25], relative risks from CPS-II study have a probability of giving conservative estimates of smoking-attributable mortality rates. Therefore, to prevent underestimation, analysis for women was excluded. Briefly, the steps included [19] are as follows : 1) The absolute age-specific lung cancer rates for 35–69 years old Canadian males of each social stratum were calculated; 2) These lung cancer rates were matched to the lung cancer rates in a mixture of smokers and non-smokers in CPS II study; 3) The proportion of lung cancer mortality attributed to smoking was used as a guide (with halving of excess mortality ratio to have conservative estimates) to estimate the smoking-attributed proportion of the mortality from UADT cancers. Considering that calculation of population-attributable fractions by conservative halving of excess risk substantially lowers the excess risk when the proportions of smokers are less but do not affect the estimates when the proportions are higher [18]; to be sure that the conservative halving does not accentuate the differences across social strata, data were analyzed both ways, with halving and without (data not shown). The results revealed that the fear was unfounded and therefore, to have conservative estimates we continued with halving of the excess risks.

Cause of death was coded according to the International Classification of Diseases (ICD-9 for 1986, 1991, and 1996 and ICD-10 for 2001). The UADT cancer sites included cancers of the lip, tongue, gums, floor of the mouth, salivary glands, tonsils, oro-pharynx, naso-pharynx, hypo-pharynx, oesophagus, larynx, glottis and epiglottis [4,6,26]. All-cause and smoking-attributable mortality rates for UADT cancers were assessed for all income quintiles. The rates assessed for the richest and richer, and middle and poorer quintiles were quite comparable (data not reported). As the number of smoking-attributable deaths was low in these quintiles, data for these quintiles were combined forming three social strata and re-analysis was done. Final analysis presented data subdividing quintiles into three strata as the highest 40%, middle 40%, and the lowest 20%. Social inequalities were measured using simple and straight forward measures, such as rate ratios and rate differences. Statistical Analysis Software (SAS 9.0) was used for data analyses. Research ethics approval was obtained from the University of Toronto.

## Results

**Figure 1:**
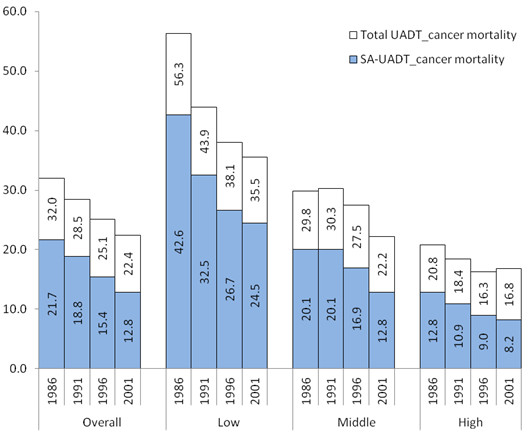
Total and smoking-attributable UADT cancer mortality rates per 100,000 males, by neighborhood income quintile.

A total of 2704 male deaths, at ages 35–69 years, due to UADT cancers were analyzed for four time points; 1986, 1991, 1996, and 2001. The present analysis showed (Figure 1) an overall mortality reduction trend due to UADT cancers among adult males in the metropolitan areas of Canada, between 1986 and 2001. Table 1 provides, estimated male death rates due to UADT cancers attributed and not attributed to smoking, which together add up to total annual death rate per 100,000 males. Between 1986 and 2001, overall UADT cancer death rates fell by 30% (32 to 22 per 100,000) and the death rates attributable to smoking fell by 41% (22 to 13 per 100,000). Men of the lowest stratum had the highest UADT cancer death rate (56 per 100,000) in 1986. By 2001, UADT cancer deaths fell by 37% (56 to 36 per 100,000) and smoking-attributable proportions fell by 42% (43 to 25 per 100,000) in the lowest stratum. In the middle stratum, UADT cancer deaths fell by 27% (30 to 22 per 100,000) and smoking-attributable proportions fell by 35% (20 to 13 per 100,000). In the highest stratum, UADT cancer deaths fell only by 19% (21 to 17 per 100,000); however, smoking-attributable proportions fell by 38% (13 to 8 per 100,000), which were comparable to other strata.

**Table 1:** Annual death rates, per 100,000 men aged 35–69 years, due to UADT cancer, attributed to smoking or not, by neighborhood income quintile, together with the stratum specific number of deaths due to lung cancer and UADT cancer and the population count, respectively

	**Overall**	**Lowest **^**ǂ **^	**Middle **^**ǂ **^	**Highest **^**ǂ **^
1986				
Yes*	21.7	42.6	20.1	12.8
No**	10.3	13.7	9.7	8.0
Total	32.0	56.3	29.8	20.8
Total UADT cancer deaths	683	258	258	167
Total lung cancer deaths	2744	830	1117	797
Population	2789815	532395	1091295	1166125
1991				
Yes*	18.8	32.5	20.1	10.9
No**	9.6	11.4	10.2	7.5
Total	28.5	43.9	30.3	18.4
Total UADT cancer deaths	680	208	306	166
Total lung cancer deaths	2881	830	1237	814
Population	3244930	593665	1283315	1367950
1996				
Yes*	15.4	26.7	16.9	9.0
No**	9.7	11.4	10.6	7.3
Total	25.1	38.1	27.5	16.3
Total UADT cancer deaths	665	188	298	179
Total lung cancer deaths	2608	696	1092	820
Population	3679785	679915	1431225	1568645
2001				
Yes*	12.8	24.5	12.8	8.2
No**	9.6	11.0	9.4	8.6
Total	22.4	35.5	22.2	16.8
Total UADT cancer deaths	676	187	269	220
Total lung cancer deaths	2487	673	1041	773
Population	4205285	778220	1628620	1798445

*UADT cancer mortality attributable to smoking, ** not attributable to smoking.

Average lung cancer rates for 35–69 year old male smokers of CPS II study was 22.2 per 100,000 and for non-smokers the rates were 6 per 100,000.

^ǂ ^ Neighborhood income quintile divided in lowest = poorest, middle = poorer + middle, and highest = richer + richest.

Table 2 provides changes in absolute and relative social inequalities in UADT cancer death rates between 1986 and 2001. In 1986, 36 more deaths per 100,000 due to UADT cancers occurred in the lowest stratum as compared to the highest, out of which 30 deaths were attributable to smoking. By 2001, absolute inequalities reduced as total rate difference between the highest and the lowest stratum fell by 47% (19 more deaths per 100,000 among the lowest stratum as compared to the highest stratum) and smoking-attributable proportions also fell by 47% (16 more deaths per 100,000 among the lowest stratum as compared to the highest stratum).

**Table 2:** Social inequality in UADT cancer mortality rate between the lowest and the highest neighborhood income quintile and the proportion of deaths attributable to smoking

	**Social inequality**		**% of smoking-attributable deaths**		
	**Rate ratio (Lowest/Highest)**	**Rate difference (Lowest-Highest)**	**Lowest **^**ǂ**^	**Highest **^**ǂ**^	**Overall**
1986					
Yes*	3.3	29.8	76	62	68
No**	1.7	5.7			
Total	2.7	35.5			
1991					
Yes*	3.0	21.6	74	59	66
No**	1.5	3.9			
Total	2.4	25.5			
1996					
Yes*	3.0	17.7	70	55	61
No**	1.6	4.1			
Total	2.3	21.8			
2001					
Yes*	3.0	16.3	69	49	57
No**	1.3	2.4			
Total	2.1	18.7			

*UADT cancer mortality attributable to smoking, ** not attributable to smoking.

^ǂ ^ Neighborhood income quintile divided in lowest = poorest, middle = poorer + middle, and highest = richer + richest.

In 1986, total death rate due to UADT cancers was 2.7 times more in the lowest stratum as compared to the highest. The proportional differences between social strata in smoking-attributed mortality were, however, more extreme at 3.3. By 2001, relative inequalities also reduced as total death rate ratios between the highest and the lowest stratum fell by 22% (2.7 in 1986 to 2.1 in 2001) and smoking-attributable death rate ratios fell by 9% (3.3 to 3.0) between 1986 and 1991 and remained constant at 3.0 from 1991 to 2001.

## Discussion

This is a descriptive analysis of trends in UADT cancer adult male mortality rates attributed to smoking among neighborhood income quintiles in urban Canada. The analysis reveals that between 1986 and 2001 UADT cancer mortality fell in all quintiles and the reductions were comparable in all quintiles. Within the quintiles, reduction in smoking-attributable proportions of UADT cancer deaths were more pronounced.

According to the Peto method, the prevalence of smoking in the study population was estimated indirectly from lung cancer death rates of the population [18]. This indirect method substitutes observed current exposure of smoking estimates with prevalence of smoking that is considered necessary for causing the current lung cancer mortality burden [27]. For most smoking-related outcomes, the current burden of disease is largely influenced by the past smoking exposure in the population [28,29]. The prevalence estimates calculated through this method avoids the potential error resulting from the lag time between population changes in smoking prevalence and the resulting change in disease outcome [27].

Excess risks were arbitrarily halved to calculate smoking-attributable fractions conservatively as some of the deaths can be attributed to other risk factors, such as alcohol and Human Papilloma virus infection. As the methods used have been acknowledged to be crude, presentation of apparently precise numbers should not be taken to suggest otherwise [18]. The statistical significance of the observed trends in smoking-attributable mortality rates were also not assessed using any method like weighted regression analysis, as the motive was to look at the trends of these rates in general in different social strata of Canada. The major pattern is, however, clear that smoking-attributable UADT cancer mortality is reducing among all social strata of Canada. This is in consensus with steady declines in male smoking prevalence (15 years and above) in Canada over the last five decades; the rates reduced from 61% in 1965 to 20% in 2010 [30]. The trends observed here are in agreement with a study done by Gupta et al. in Canada, and the US, which stated that the incidence of UADT cancers reduced between 1984 and 2001 [6]. A possible explanation of this reduction can be tobacco control policies (for example significant increase in tobacco taxes in 1980’s and early 1990’s) which were implemented at that time period affecting the smoking prevalence. The results observed are also in consensus with Reid et al., who observed smoking prevalence among different social strata in Canada, 1999–2006, also revealed absolute reductions in daily smoking and cigarettes consumed per day in both the highest and the lowest social strata [16].

For the analysis, the relative risk of smoking-attributable UADT cancer mortality was considered the same across all quintiles; however, there are many factors other than smoking that differ between quintiles [31] and as smoking interacts with other risk factors [32], the hazard for the individual smoker must also be expected to be different across various quintiles. However, Thun et al., for a US study, determined that smoking-attributable deaths reduced by just 1% per year after adjusting for other factors like education, occupation, race, alcohol consumption, and various dietary factors, in addition to age and sex [33].

Because of lack of any large national representative mortality study, usage of relative risks based on CPS II study was another limitation; however, the mortality risks of CPS II study for various diseases have been quite acceptable in the Canadian context. Another limitation was the use of neighborhood level information, instead of family or individual, and applying to individuals, which forces consideration of the ecological fallacy. However, past studies have argued for the validity of using income quintiles as a proxy for individual socioeconomic status [20,22].

Mackenback and Kunst, in 1997, presented a framework for measuring the magnitude of socio-economic inequalities in health, according to which, simple and straightforward measures are more useful in informing policy makers [34]. Relative Index of Inequality (RII), Slope Index of Inequality (SII), and Concentration Index on the other hand have a complex interpretation and can easily lead to misunderstandings [34]. Therefore, we used rate ratios and rate differences to depict social inequalities.

Although the methods of estimation used are indirect and have some limitations, the uncertainties inherent in these methods affect all social strata similarly; therefore, cannot account for overestimation of the differences observed between social strata in smoking-attributed mortality.

## Conclusion

Between 1986 and 2001, the social inequalities in smoking-attributable UADT cancer mortality rates among adult males (35–69 years) in Canada have narrowed. In context of implementation of different tobacco control policies in the past two decades, assessment of smoking-attributable rates of more recent years would be of interest to confirm the trends observed.

## Abbreviations

UADT: Upper aero-digestive tract.

## Competing interest

We declare that we have no financial or non-financial competing interest.

## Authors’ contributions

SS, CQ, and PJ planned the paper. SS and PJ obtained data. SS performed all statistical analyses. All authors participated in interpreting the results and contributed equally in writing the manuscript. All authors read and approved the final manuscript.

## Pre-publication history

The pre-publication history for this paper can be accessed here:

http://www.biomedcentral.com/1471-2458/13/328/prepub
